# The impact of 12 modifiable lifestyle behaviours on depressive and anxiety symptoms in middle adolescence: prospective analyses of the Canadian longitudinal COMPASS study

**DOI:** 10.1186/s12966-023-01436-y

**Published:** 2023-04-17

**Authors:** Julia Dabravolskaj, Paul J. Veugelers, Angelica Amores, Scott T. Leatherdale, Karen A. Patte, Katerina Maximova

**Affiliations:** 1 Edmonton Clinic Health Academy, School of Public Health, University of Alberta, 11405 87 Ave NW, Edmonton, AB T6G 1C9 Canada; 2 School of Public Health Sciences, University of Waterloo, Waterloo, ON Canada; 3 Faculty of Applied Health Sciences, Brock University, St. Catharines, ON Canada; 4 MAP Centre for Urban Health Solutions, Li Ka Shing Knowledge Institute, St. Michael’s Hospital, Toronto, ON Canada; 5 Dalla Lana School of Public Health, University of Toronto, Toronto, ON Canada

**Keywords:** Adolescence, Youth, Lifestyle behaviours, Mental health, Depressive symptoms, Anxiety symptoms

## Abstract

**Background:**

Unhealthy lifestyle behaviours are becoming increasingly common and might contribute to the growing burden of mental disorders in adolescence. We examined the associations between a comprehensive set of lifestyle behaviours and depression and anxiety in middle adolescents.

**Methods:**

School-based survey responses were collected from 24,274 Canadian high school students at baseline and 1-year follow-up (average age 14.8 and 15.8 years, respectively). Using linear mixed-effects models, we examined prospective associations of adherence to recommendations for vegetables and fruit, grains, milk and alternatives, meat and alternatives, sugar-sweetened beverages [SSB], physical activity, screen time, sleep, and no use of tobacco, e-cigarettes, cannabis, and binge drinking at baseline with the depressive and anxiety symptoms (measured by CESD-R-10 and GAD-7 scales, respectively) at follow-up.

**Results:**

Adherence to recommendations was low overall, particularly for vegetables and fruit (3.9%), grains (4.5%), and screen time (4.9%). Students adhering to individual recommendations, particularly for meat and alternatives, SSB, screen time, sleep, and no cannabis use, at baseline had lower CESD-R-10 and GAD-7 scores at follow-up. Adhering to every additional recommendation was associated with lower CESD-R-10 (β=-0.15, 95% CI -0.18, -0.11) and GAD-7 scores (β=-0.10, 95% CI -0.14, -0.07) at follow-up. Assuming cumulative impact, this might translate into 7.2- and 4.8-point lower CESD-R-10 and GAD-7 scores, respectively, among students adhering to 12 vs. 0 recommendations over four years of high school.

**Conclusions:**

The results highlight the preventive potential of population-based approaches promoting healthy lifestyle behaviours, particularly those with the lowest prevalence, as a strategy to improve mental health in adolescence.

**Supplementary Information:**

The online version contains supplementary material available at 10.1186/s12966-023-01436-y.

## Introduction

Mental health problems are becoming increasingly common among adolescents in Canada, as reflected in the increasing rates of related healthcare visits even before the COVID-19 pandemic. For example, compared to stable rates of healthcare visits due to physical health conditions, there were 53% more emergency department and 74% more inpatient visits due to mental health problems (most commonly anxiety, mood, and substance use disorders) in 15-17-year-olds between 2006/07 and 2013/14 [1]. Pandemic-related disruptions have further exacerbated the mental health burden in adolescents, with noted increases in the proportion of mental health-related hospitalizations and the use of mood and anxiety medications. [2] This underscores the urgent need for population-based health promotion and primary prevention [3] that, in the absence of effective treatment options, are a crucial strategy to curb the mental health burden.

The evidence on the detrimental impact that substance use (i.e., tobacco smoking, binge drinking, [4] vaping, [5] cannabis use [6]), lack of physical activity, excess screen time, [7] or poor sleep [8] each individually have on the development of mental health problems is strong, and the importance of healthy diet is emerging too. [9, 10] Studies generally focus on the impact of individual lifestyle behaviours, but this does not augment our understanding of the intricate relationships among lifestyle behaviours which tend to cluster among adolescents, [11–13] and how these intertwined behaviours may be linked to mental health. Several studies quantified associations between belonging to different clusters of lifestyle behaviours and mental health outcomes in adolescents. [14, 15] While interesting, such studies often examine different combinations of a limited number of lifestyle behaviours and/or assess them using different scales, thus making results across studies difficult to compare. Operationalizing lifestyle behaviours in terms of adherence to existing evidence-based recommendations (which remain mostly unchanged over the years and comparable across countries) can help circumvent these challenges. Additionally, results of studies examining adherence to lifestyle recommendations in relation to mental health outcomes can be easier to understand by public health professionals and thus conducive to being implemented in practice.

To our knowledge, there is only one prospective study [16] that has estimated the effect of overall adherence to nine lifestyle recommendations (i.e., vegetables and fruit, grains, milk and alternatives, meat and alternatives, added sugar, saturated fat, sleep, screen time, physical activity) on the risk of developing internalizing and externalizing mental disorders in *early adolescence* (i.e., ages 10–14). However, as adolescents mature, they gain more independence, their social relationships become more complex and influential, [17] and as a result they often adopt new unhealthy behaviours, particularly substance use behaviours, often initiated around the age of 15. [18, 19] In fact, 30% of middle adolescents (i.e., 14–17 years) reported binge drinking, e-cigarette smoking (vaping), and drug use in 2018/19 [20]. Given this high prevalence of substance use behaviours, it is imperative to evaluate a wide range of lifestyle behaviours in relation to mental health outcomes in middle adolescence. To date, no Canadian study has examined the extent to which the co-occurrence of a full range of lifestyle behaviours (including substance use) may relate to mental illness in this age group. Therefore, the goal of this study is two-fold: (1) to examine individual and overall adherence to 12 lifestyle recommendations (i.e., five recommendations for diet and one each for physical activity, screen time, sleep, and no tobacco smoking, vaping, cannabis use, and binge drinking), and (2) to assess the relationship between adherence to these recommendations and the severity of depressive and anxiety symptoms among Canadian middle adolescents. We hypothesized that both individually and in combination, healthier lifestyle behaviours are prospectively associated with better mental health in middle adolescence.

## Methods

The Cannabis, Obesity, Mental health, Physical activity, Alcohol, Smoking, and Sedentary behaviour (COMPASS) is a large longitudinal study that annually collects survey data on an extensive range of lifestyle behaviours from more than 65,000 grade 9–12 students (age 13–18 years) recruited through a convenience sample of 122 secondary schools in Alberta, British Columbia, Ontario, and Québec, Canada. [21] Using an active-information passive-consent parental permission protocol, students complete an anonymous COMPASS questionnaire at school during class time. COMPASS procedures are available elsewhere. [21] For this study, we linked [22] participants’ responses from 2017/18 (average age of participants 14.8 [SD = 1.2] years old) to the same participants’ responses from 2018/19 (average age 15.8 [SD = 1.2] years old). Records of grade 12 participants and those who changed schools between 2017/18 and 2018/19 were not linked: out of 66,434 participants from 122 schools in 2017/18, the linked sample comprised records of 29,022 participants from 116 schools. Analyses were based on a subsample of 24,274 participants, with data available for all variables listed below. Response rates in 2017/18 and 2018/19 were 81.8% and 84.2%, respectively, with non-response being mainly due to absenteeism or scheduled spare time during data collection. [23].

### Measures

The Center for Epidemiologic Studies Depression Scale Revised-10 (CESD-R-10) [24] is a 10-item self-report scale that queries participants about the frequency of symptoms of unipolar depression in the last seven days (none or < 1 day; 1–2 days; 3–4 days; and 5–7 days). The Generalized Anxiety Disorder-7 (GAD-7) scale [25] is a 7-item self-report scale that asks participants about the frequency of generalized anxiety disorder symptoms in the last two weeks (not at all; several days; over half the days; and nearly every day). Both scales demonstrated strong psychometric properties in adolescents. [26, 27] On both scales, items are scored from 0 to 3. Scores are then summed, with the final scores ranging from 0 to 30 for CESD-R-10 and 0 to 21 for GAD-7 scales. Higher scores on both scales indicate greater severity of symptoms. For both scales, missing values on up to two items were imputed for 5168 participants. [28–30].

Adherence to established recommendations [31–35] was conceptualized as both individual and overall adherence to recommendations for: vegetables and fruit, grains, milk and alternatives, meat and alternatives, sugar-sweetened beverages (SSB), physical activity, screen time, sleep, and no use of tobacco, e-cigarettes, cannabis, and binge drinking. Participants reported the number of servings (up to nine or more) of *vegetables and fruit, grain products, milk and alternatives*, and *meat and alternatives* they consumed the previous day. [36] Participants reported the number of days in a usual school week when they consumed sugar-sweetened beverages, high-energy drinks, and coffee or tea with sugar, which were combined into a *sugar-sweetened beverages* composite variable (SSB). Participants reported the number of minutes of *moderate-to-vigorous physical activity* (MVPA) on each day in the past week; the number of hours and minutes per day they usually spend watching/streaming TV shows or movies, playing video/computer games, talking on the phone, surfing the Internet, and texting, messaging, emailing, which were summed to obtain total daily *screen time* [37]; and the number of hours and minutes per day they usually *sleep.* Participants reported the past-30-days frequency of smoking one or more *cigarettes* and *e-cigarettes* (vaping) (none, 1, 2–3, 4–5, 6–10, 11–20, 21–29, 30 days) and the past-12-months frequency of *cannabis use* (never, used but not in the past 12 months, less than once/month, once/month, 2–3 times/month, once/week, 2–3 times/week, 4–6 times/week, and every day) and of having five or more drinks of alcohol on one occasion (i.e., *binge drinking*) (never, not in the past 12 months, less than once/month, once/month, 2–3 times/month, once/week, 2–5 times/week, daily or almost daily). Participants that never tried cigarettes, cannabis use, and binge drinking were considered as those meeting recommendations. Participants’ responses to questions pertaining to each lifestyle behaviour were assigned 0 points if a recommendation was not met and 1 point if it was met (Table 1).

**Table 1:** Existing recommendations for 12 lifestyle behaviours in adolescents in Canada

Lifestyle behaviour	Recommendation met if:†
Vegetables and fruit, servings/day	females ≥ 7, males ≥ 8
Grain products, servings/day	females ≥ 6, males ≥ 7
Milk and alternatives, servings/day	females and males ≥ 3
Meat and alternatives, servings/day	females ≥ 2, males ≥ 3
SSB, servings/day	0
MVPA, minutes/day	≥ 60
Screen time, hours/day	< 2
Sleep, hours/day	8–10
Tobacco smoking	no tobacco smoking ever or in the past 30 days
Vaping	no vaping in the past 30 days
Cannabis use	no cannabis use ever or in the past 12 months
Binge drinking	no binge drinking ever or in the past 12 months

SSB: sugar-sweetened beverages; MVPA: moderate-to-vigorous physical activity.

†The Canada’s Food Guide (2011) was used to determine adherence to recommendations for vegetables and fruit, grain products, milk and alternatives, meat and alternatives [31]; the Canada’s Food Guide (2021) – for SSB; [38] the Canadian 24-hour movement guidelines – for MVPA, screen time and sleep. [32] Substance use behaviours are not recommended. [33–35].

Points for all lifestyle behaviours were then summed to create a composite score ranging from 0 to 12, with higher scores indicating higher overall adherence to lifestyle recommendations and thus a healthier lifestyle. Additionally, based on the composite score, participants were categorized into those having very unfavourable (meeting ≤ 3 recommendations), unfavourable (4–6), intermediate (7–9), or favourable (10–12) lifestyles.

### Covariates

A-priori-defined confounders were *age* (years), *sex* (female, male), *ethnicity* (White, Black, Hispanic, Asian, Other/Mixed), *school area urban class* (rural area and small [population of 1,000–29,999], medium [30,000–99,999], and large urban [≥ 100,000] population centers, defined by the 2016 Canadian Census [39]), *school-area median household income* ($20,000-$40,000, $40,001-$60,000, $60,001-$80,000, and more than $80,000, derived from the first three letters of each school’s postal code), *weight status* (derived from self-reported height and weight and categorized into underweight, normal weight, overweight, obese, and missing according to age- and sex-specific cut-off points [40]), *weight perception* (very or slightly underweight, about the right weight, slightly or very overweight), and *weight loss attempts* (positive responses to the statement “[I am] trying to lose weight”). The latter three covariates were included to account for the potentially confounding effect of these variables on associations between lifestyle behaviours, particularly diet and physical activity, and mental health outcomes.

### Data analyses

We used Student’s *t*-test and analysis of variance (ANOVA) tests to assess differences in mean CESD-R-10 and GAD-7 scores. We estimated 1-year prospective associations between individual and overall adherence to lifestyle recommendations at baseline and the severity of depressive and anxiety symptoms at 1-year follow-up. Univariate and multivariable linear mixed-effects models (LMMs) were used to account for the nested data structure (i.e., students in schools). Multivariable models were first adjusted for age, ethnicity, school area urban class, school-area median household income, weight status, weight perception, weight loss attempts, and mental health at baseline (CESD-R-10 and GAD-7 scores, as appropriate) (Model 1). Next, these models were repeated while being mutually adjusted for other lifestyle behaviours (Model 2) to quantify the *individual effect* of separate lifestyle behaviours on the severity of depressive and anxiety symptoms in those meeting vs. not meeting recommendations. In this study, we consider lifestyle behaviours as competing exposures and their relationships with mental health outcomes to be similarly confounded. To compare the *combined effect* of all 12 lifestyle behaviours on the severity of depressive and anxiety symptoms in those with unfavourable, intermediate, and favourable lifestyles (i.e., meeting 4–6, 7–9, and 10–12 recommendations, respectively) vs. very unfavourable lifestyle (i.e., meeting ≤ 3 recommendations), we ran models adjusting for the same covariates as Model 1 described above. Finally, we ran the same models with the number of recommendations met as the independent variable to quantify the effect of adhering to each additional recommendation on the severity of depressive and anxiety symptoms. The final models were sex-stratified since lifestyle behaviours [41, 42] and mental health [43] are sex-patterned. Missing baseline values for age, sex, and ethnicity were imputed based on the available information (e.g., age reported in 2018/19 minus one, and sex and ethnicity – same as in 2018/19). All models were adjusted for confounders (and confounding is a causal concept [44]), therefore we use causal language throughout the manuscript for transparency and clarity. [45] Cross-sectional associations at baseline are reported in Supplementary materials (Tables S1 and S2) but are not discussed in this paper. Analyses were performed using SAS 9.4 (SAS Institute, Cary, NC).

## Results

Participant characteristics are provided in Table 2: 54.4% were female, 71.6% identified as white, 55.9% resided in large population centres, and 42.2% attended schools located in areas of higher median household income. More than half of the participants were classified as normal weight. Compared to males, females more often perceived themselves as normal weight (58.4% vs. 52.4%) and reported weight loss attempts (41.1% vs. 21.8%).

**Table 2:** Characteristics of participants (n = 24,274) in the COMPASS study at baseline (2017/18 school year)

	Total (n = 24,274)	Females (n = 13,204)	Males (n = 11,070)
*Age*, mean (SD)	14.8 (1.2)	14.8 (1.2)	14.8 (1.2)
*Sex, %*			
Females	54.4	N/A	N/A
Males	45.6	N/A	N/A
*Ethnicity, %*			
White	71.6	71.2	72.1
Black	2.6	2.4	2.8
Asian	10.6	10.7	10.5
Latin American/Hispanic	1.9	1.9	1.9
Other/mixed	13.3	13.8	12.7
*Province, %*			
Ontario	47.1	46.3	48.1
Alberta	5.1	4.9	5.2
British Columbia	13.9	13.6	14.4
Quebec	33.9	35.2	32.3
*School area urban class, %*			
Rural area	0.6	0.6	0.5
Small urban population centres	31.6	31.0	32.2
Medium urban population centres	11.9	11.9	12.1
Large urban population centres	55.9	56.5	55.2
*School-area median income (CAD), %*			
20,000 to 40,000	5.8	6.0	5.5
40,001 to 60,000	31.4	32.2	30.4
60,001 to 80,000	42.2	41.6	42.7
80,000+	20.6	20.2	21.4
*Weight status, %*			
Underweight	1.9	1.7	2.1
Normal weight	55.7	58.4	52.4
Overweight	12.1	10.5	14.0
Obese	5.4	3.8	7.2
Missing	24.9	25.6	24.3
*Weight perception, %*			
Underweight	16.3	11.5	21.9
About the right weight	60.8	63.5	57.7
Overweight	22.9	25.0	20.4
*Weight loss attempts, %*			
Yes	32.3	41.1	21.8
No	67.7	58.9	78.2
*Adherence to individual recommendations for, %*			
Vegetables and fruit	3.9	4.6	3.1
Grain products	4.5	3.8	5.3
Milk and alternatives	36.4	28.9	45.5
Meat and alternatives	55.4	65.0	43.9
SSB	28.9	30.8	26.7
MVPA	38.5	31.5	46.8
Screen time	4.9	5.8	3.8
Sleep	48.3	46.4	50.7
No tobacco smoking	93.2	94.3	91.9
No vaping	83.5	86.0	80.6
No cannabis use	86.4	87.1	85.6
No binge drinking	75.0	74.9	75.1
*Number of recommendations met, %*			
0	0.4	0.2	0.2
1	1.4	0.8	0.7
2	3.3	1.8	1.5
3	6.1	3.3	2.7
4	12.8	6.8	6.0
5	22.0	11.9	10.1
6	24.0	13.3	10.7
7	17.7	9.8	7.9
8	8.6	4.6	4.0
9	3.0	1.6	1.4
10	0.6	0.3	0.3
11	0.1	0.0	0.1
12	0.0	0.0	0.0
*Number of recommendations met, mean (SD)*	5.6 (1.7)	5.6 (1.7)	5.6 (1.8)
Lifestyle based on overall adherence, %**			
Very unfavourable	11.1	11.1	11.2
Unfavourable	58.9	59.0	58.7
Intermediate	29.3	29.3	29.2
Favourable	0.7	0.6	0.9
*CESD-R-10 score, mean (SD)*	8.0 (5.7)	9.1 (6.1)	6.7 (4.8)
*GAD-7 score, mean (SD)*	5.9 (5.3)	7.2 (5.6)	4.3 (4.5)

CAD: Canadian dollar; MVPA: moderate-to-vigorous physical activity; SD: standard deviation; SSB: sugar-sweetened beverages

**Those meeting 3 or less recommendations were classified as having very unfavourable, 4–6 – unfavourable, 7–9 – intermediate, and 10–12 – favourable lifestyles

Only 3.9% of participants met recommendations for vegetables and fruit, 4.5% for grains, and 4.9% for screen time. Close to one-third (28.9%) met recommendations for SSB, 36.4% for milk and alternatives, and 38.5% for MVPA (Table 2). Recommendations for sleep and meat and alternatives were met by 48.3% and 55.4%, respectively. Three-quarters of participants reported no binge drinking, 83.5% no vaping, 86.4% no cannabis use, and 93.2% no tobacco smoking. On average, participants in this study met 5.6 recommendations, while almost none of the participants met all 12 recommendations. Overall, 70.0% of participants were classified as having very unfavourable or unfavourable lifestyles, while 29.3% had intermediate and 0.7% favourable lifestyles. No notable sex differences were observed in individual or overall adherence to 12 lifestyle recommendations.

At baseline and follow-up, CESD-R-10 scores were 8.03 (SD = 5.70) and 8.92 (SD = 5.99) and GAD-7 scores 5.85 (SD = 5.30) and 6.47 (SD = 5.50), respectively (Table 2). At baseline, CESD-R-10 and GAD-7 scores were lower among participants who followed recommendations for individual lifestyle behaviours, except for vegetables and fruit, and grains (Table 3). Those who were meeting recommendations for vegetables and fruit, and grains had similar CESD-R-10 scores and higher GAD-7 scores than those not meeting these recommendations. Participants with a very unfavourable lifestyle (i.e., meeting ≤ 3 recommendations) had generally higher CESD-R-10 and GAD-7 scores (10.60 [SD = 6.42] and 7.89 [SD = 5.89], respectively) compared to those with favourable lifestyle (5.49 [SD = 4.76] and 3.87 [SD = 4.90], respectively). This pattern was particularly pronounced in females.

**Table 3:** CESD-R-10 and GAD-7 scores by adherence to lifestyle recommendations at baseline (mean age 14.8 years)

	Depressive symptoms (CESD-R-10 score)			Anxiety symptoms (GAD-7 score)			
	Total	Females	Males	Total	Females	Males	
	Mean (SD)	Mean (SD)	Mean (SD)	Mean (SD)	Mean (SD)	Mean (SD)	
*Adherence to individual recommendations for*:							
Vegetables and fruit							
yes	7.94 (6.09)	8.71 (6.28)	6.56 (5.48)	6.16 (5.79)	7.18 (5.92)	4.31 (5.06)	
no	8.04 (5.68)	9.15 (6.11)	6.72 (4.81)	5.83 (5.28)	7.17 (5.54)	4.27 (4.47)	
Grain products							
yes	8.00 (5.71)	9.14 (6.11)	7.03 (5.15)	5.99 (5.49)	7.41 (5.77)	**4.77 (4.93)**	
no	8.03 (5.70)	9.13 (6.12)	6.70 (4.81)	5.84 (5.29)	7.16 (5.55)	**4.24 (4.46)**	
Milk and alternatives							
yes	**7.39 (5.46)**	**8.59 (6.10)**	**6.47 (4.73)**	**5.28 (5.12)**	**6.82 (5.59)**	**4.11 (4.40)**	
no	**8.40 (5.80)**	**9.35 (6.12)**	**6.92 (4.90)**	**6.17 (5.37)**	**7.31 (5.54)**	**4.40 (4.56)**	
Meat and alternatives							
yes	**7.81 (5.54)**	**8.55 (5.82)**	**6.50 (4.74)**	5.79 (5.15)	**6.71 (5.32)**	**4.16 (4.39)**	
no	**8.31 (5.87)**	**10.21 (6.50)**	**6.89 (4.89)**	5.92 (5.47)	**8.02 (5.88)**	**4.36 (4.56)**	
SSB							
yes	**7.30 (5.61)**	**8.13 (6.01)**	**6.15 (4.77)**	**5.19 (5.09)**	**6.26 (5.36)**	**3.72 (4.28)**	
no	**8.33 (5.71)**	**9.57 (6.12)**	**6.93 (4.84)**	**6.11 (5.36)**	**7.57 (5.60)**	**4.47 (4.54)**	
MVPA							
yes	**7.91 (5.69)**	**9.51 (6.24)**	**6.61 (4.83)**	5.77 (5.44)	**7.69 (5.78)**	4.23 (4.60)	
no	**8.11 (5.70)**	**8.95 (6.06)**	**6.82 (4.83)**	5.89 (5.21)	**6.93 (5.44)**	4.31 (4.39)	
Screen time							
yes	**6.15 (4.95)**	**6.70 (5.20)**	**5.15 (4.31)**	**4.68 (5.00)**	**5.49 (5.28)**	**3.20 (4.08)**	
no	**8.13 (5.72)**	**9.28 (6.14)**	**6.78 (4.84)**	**5.91 (5.31)**	**7.27 (5.56)**	**4.31 (4.50)**	
Sleep							
yes	**6.73 (5.04)**	**7.66 (5.55)**	**5.71 (4.18)**	**4.82 (4.76)**	**5.95 (5.08)**	**3.59 (4.05)**	
no	**9.25 (6.00)**	**10.41 (6.30)**	**7.76 (5.22)**	**6.80 (5.59)**	**8.22 (5.74)**	**4.97 (4.80)**	
No tobacco smoking							
yes	**7.83 (5.58)**	**8.88 (5.99)**	**6.55 (4.72)**	**5.70 (5.21)**	**6.97 (5.49)**	**4.14 (4.38)**	
no	**10.78 (6.57)**	**13.32 (6.76)**	**8.63 (5.55)**	**7.84 (6.02)**	**10.33 (5.81)**	**5.75 (5.36)**	
No vaping							
yes	**7.78 (5.59)**	**8.76 (5.99)**	**6.52 (4.74)**	**5.66 (5.21)**	**6.88 (5.47)**	**4.11 (4.40)**	
no	**9.33 (6.05)**	**11.38 (6.42)**	**7.55 (5.09)**	**6.78 (5.63)**	**8.94 (5.79)**	**4.93 (4.77)**	
No cannabis use							
yes	**7.66 (5.50)**	**8.65 (5.90)**	**6.46 (4.69)**	**5.56 (5.15)**	**6.79 (5.41)**	**4.06 (4.35)**	
no	**10.38 (6.36)**	**12.36 (6.59)**	**8.25 (5.34)**	**7.65 (5.87)**	**9.67 (5.88)**	**5.50 (5.04)**	
No binge drinking							
yes	**7.63 (5.50)**	**8.56 (5.92)**	**6.51 (4.70)**	**5.50 (5.16)**	**6.70 (5.45)**	**4.08 (4.38)**	
no	**9.24 (6.11)**	**10.83 (6.38)**	**7.33 (5.14)**	**6.87 (5.58)**	**8.57 (5.64)**	**4.85 (4.76)**	
Lifestyle based on overall adherence**							
Very unfavourable	**10.60 (6.42)**	**12.47 (6.61)**	**8.38 (5.43)**	**7.89 (5.89)**	**9.84 (5.80)**	**5.62 (5.13)**	
Unfavourable	**8.34 (5.66)**	**9.47 (6.04)**	**6.98 (4.84)**	**6.05 (5.29)**	**7.40 (5.51)**	**4.42 (4.50)**	
Intermediate	**6.52 (5.01)**	**7.27 (5.42)**	**5.61 (4.30)**	**4.71 (4.77)**	**5.73 (5.10)**	**3.48 (4.02)**	
Favourable	**5.49 (4.76)**	**6.03 (5.15)**	**5.06 (4.39)**	**3.87 (4.90)**	**4.63 (5.21)**	**3.26 (4.57)**	

MVPA: moderate-to-vigorous physical activity; SD: standard deviation; SSB: sugar-sweetened beverages

*Bolded are values with p-values < 0.05

**Those meeting 3 or less recommendations were classified as having very unfavourable, 4–6 – unfavourable, 7–9 – intermediate, and 10–12 –favourable lifestyles

When considered individually, adherence to lifestyle recommendations, particularly for meat and alternatives, SSB, screen time, sleep, and no cannabis use at baseline was associated with lower CESD-R-10 and GAD-7 scores at follow-up (Table 4). Sex-based subgroup analyses revealed some differences. Females adhering to recommendations for meat and alternatives, SSB, screen time, and sleep had lower severity of depressive and anxiety symptoms, while the opposite was observed for MVPA: contrary to expectations, adherence to the individual recommendation for MVPA was associated with higher severity of depressive symptoms. Males adhering to recommendations for SSB, MVPA, screen time, sleep, and cannabis use had lower severity of depressive and particularly anxiety symptoms.

**Table 4:** Associations of adherence to 12 lifestyle recommendations at baseline with CESD-R-10 and GAD-7 scores at 1-year follow-up

	Univariate	Multivariable*			
		Model 1	Model 2		
	Total	Total	Total	Females	Males
	β (95% CI)	β (95% CI)	β (95% CI)	β (95% CI)	β (95% CI)
*Adherence to individual recommendations for*:	**Depressive symptoms**				
Vegetables and fruit	-0.29 (-0.68, 0.10)	**-0.40 (-0.71, -0.09)**	-0.26 (-0.57, 0.06)	-0.17 (-0.59, 0.24)	-0.45 (-0.95, 0.06)
Grain products	-0.35 (-0.71, 0.02)	-0.19 (-0.49, 0.10)	-0.06 (-0.36, 0.24)	-0.02 (-0.47, 0.44)	-0.11 (-0.51, 0.28)
Milk and alternatives	**-0.89 (-1.05, -0.73)**	-0.09 (-0.22, 0.04)	-0.03 (-0.16, 0.10)	-0.03 (-0.23, 0.16)	-0.04 (-0.22, 0.13)
Meat and alternatives	**-0.30 (-0.45, -0.14)**	**-0.28 (-0.41, -0.16)**	**-0.24 (-0.37, -0.11)**	**-0.38 (-0.56, -0.19)**	-0.10 (-0.28, 0.08)
SSB	**-0.76 (-0.93, -0.59)**	**-0.28 (-0.42, -0.15)**	**-0.20 (-0.34, -0.06)**	-0.19 (-0.38, 0.00)	**-0.22 (-0.42, -0.03)**
MVPA	**-0.46 (-0.62, -0.31)**	0.05 (-0.08, 0.18)	0.08 (-0.05, 0.21)	**0.31 (0.12, 0.50)**	**-0.19 (-0.36, -0.01)**
Screen time	**-1.79 (-2.14, -1.44)**	**-0.76 (-1.04, -0.48)**	**-0.65 (-0.93, -0.36)**	**-0.76 (-1.13, -0.39)**	-0.41 (-0.86, 0.04)
Sleep	**-1.98 (-2.13, -1.83)**	**-0.49 (-0.62, -0.37)**	**-0.45 (-0.58, -0.33)**	**-0.53 (-0.71, -0.34)**	**-0.38 (-0.56, -0.20)**
No tobacco smoking	**-1.89 (-2.20, -1.59)**	-0.24 (-0.48, 0.01)	-0.03 (-0.31, 0.26)	0.02 (-0.40, 0.45)	-0.03 (-0.41, 0.35)
No vaping	**-1.02 (-1.23, -0.81)**	**-0.19 (-0.36, -0.02)**	-0.04 (-0.24, 0.16)	0.10 (-0.18, 0.39)	-0.16 (-0.43, 0.11)
No cannabis use	**-1.81 (-2.03, -1.59)**	**-0.36 (-0.55, -0.18)**	**-0.29 (-0.51, -0.07)**	-0.23 (-0.55, 0.09)	**-0.37 (-0.68, -0.06)**
No binge drinking	**-1.09 (-1.26, -0.91)**	-0.12 (-0.27, 0.03)	0.06 (-0.12, 0.23)	0.06 (-0.18, 0.31)	0.06 (-0.19, 0.30)
*Adherence to individual recommendations for*:	**Anxiety symptoms**				
Vegetables and fruit	0.32 (-0.03, 0.68)	-0.07 (-0.35, 0.21)	0.01 (-0.27, 0.29)	0.00 (-0.36, 0.37)	-0.02 (-0.48, 0.44)
Grain products	-0.07 (-0.41, 0.26)	0.02 (-0.24, 0.29)	0.07 (-0.20, 0.34)	-0.04 (-0.45, 0.36)	0.15 (-0.20, 0.50)
Milk and alternatives	**-0.71 (-0.85, -0.57)**	0.04 (-0.07, 0.16)	0.08 (-0.04, 0.19)	0.09 (-0.09, 0.26)	0.06 (-0.10, 0.22)
Meat and alternatives	0.09 (-0.05, 0.23)	**-0.19 (-0.30, -0.08)**	**-0.19 (-0.30, -0.07)**	**-0.27 (-0.44, -0.11)**	-0.08 (-0.25, 0.08)
SSB	**-0.66 (-0.81, -0.51)**	**-0.26 (-0.38, -0.14)**	**-0.19 (-0.31, -0.07)**	**-0.21 (-0.38, -0.04)**	**-0.19 (-0.37, -0.01)**
MVPA	**-0.42 (-0.56, -0.27)**	0.09 (-0.03, 0.20)	0.10 (-0.02, 0.21)	**0.29 (0.13, 0.46)**	-0.11 (-0.27, 0.05)
Screen time	**-1.14 (-1.46, -0.82)**	**-0.64 (-0.89, -0.39)**	**-0.55 (-0.80, -0.29)**	**-0.60 (-0.93, -0.27)**	**-0.46 (-0.86, -0.06)**
Sleep	**-1.53 (-1.67, -1.39)**	**-0.40 (-0.51, -0.29)**	**-0.37 (-0.49, -0.26)**	**-0.32 (-0.49, -0.16)**	**-0.46 (-0.62, -0.30)**
No tobacco smoking	**-1.36 (-1.63, -1.09)**	-0.15 (-0.37, 0.07)	0.10 (-0.15, 0.35)	0.19 (-0.18, 0.56)	0.02 (-0.31, 0.36)
No vaping	**-0.82 (-1.01, -0.64)**	**-0.23 (-0.38, -0.08)**	-0.13 (-0.31, 0.04)	-0.12 (-0.38, 0.13)	-0.08 (-0.32, 0.16)
No cannabis use	**-1.47 (-1.67, -1.26)**	**-0.34 (-0.50, -0.17)**	**-0.29 (-0.49, -0.10)**	-0.15 (-0.44, 0.13)	**-0.49 (-0.77, -0.22)**
No binge drinking	**-0.98 (-1.14, -0.82)**	-0.09 (-0.23, 0.04)	0.11 (-0.05, 0.26)	0.15 (-0.06, 0.37)	0.07 (-0.15, 0.28)

β: unstandardized regression coefficient; 95% CI: 95% confidence interval; MVPA: moderate-to-vigorous physical activity; SSB: sugar-sweetened beverages

*In linear mixed-effects models, Model 1 was adjusted for age, ethnicity, weight status, weight perception, weight loss attempts, school- area median household income, school area urban class, and mental health at baseline (CESD-R-10 and GAD-7 scores, as appropriate). Model 2 was adjusted for the covariates listed for Model 1 and mutually adjusted for all other lifestyle behaviours. Not meeting recommendations was the reference category in all analyses presented in Table 4. 95% CIs that do not include the null value are bolded

As for the combined effect, for every additional lifestyle recommendation met, females (β=-0.13, 95% CI -0.18, -0.08) and males (β=-0.17, 95% CI -0.22, -0.12) had lower CESD-R-10 scores at follow-up. Compared to those with the very unfavourable lifestyle (i.e., meeting ≤ 3 recommendations), females and males with the favourable lifestyle (i.e., meeting 10–12 recommendations) had lower CESD-R-10 scores (Table 5: β=-1.15, 95% CI -2.28, -0.02 and β=-1.35, 95% CI -2.28, -0.41, respectively) and males with unfavourable and intermediate (i.e., meeting 7–9 recommendations) lifestyles had lower GAD-7 scores (β=-0.29, 95% CI -0.54, -0.03 and β=-0.65, -0.93, -0.38, respectively) at follow-up. Given that students spend four years in high school and assuming the homogeneity and additivity of the effect of lifestyle behaviours on mental health outcomes throughout high school, these estimates may add up to 4.6- and 5.4-point lower CESD-R-10 scores in females and males with the favourable lifestyle and up to 1.16- and 2.6-point lower GAD-7 scores in males with the unfavourable and intermediate lifestyles, compared to those with the very unfavourable lifestyle. Cross-sectional estimates (Table S1 and S2) were more pronounced than those in prospective analyses.

**Table 5:** Associations of overall adherence to 12 lifestyle recommendations at baseline with CESD-R-10 and GAD-7 scores at 1-year follow-up

	Univariate	Multivariable*		
	Total	Total	Females	Males
	β (95% CI)	β (95% CI)	β (95% CI)	β (95% CI)
Lifestyle based on overall adherence**	***Depressive symptoms***			
Unfavourable	**-1.45 (-1.69, -1.20)**	-0.15 (-0.35, 0.06)	0.04 (-0.25, 0.33)	**-0.35 (-0.64, -0.07)**
Intermediate	**-2.87 (-3.14, -2.61)**	**-0.54 (-0.77, -0.32)**	**-0.33 (-0.65, -0.01)**	**-0.80 (-1.11, -0.49)**
Favourable	**-4.17 (-5.07, -3.28)**	**-1.22 (-1.95, -0.49)**	**-1.15 (-2.28, -0.02)**	**-1.35 (-2.28, -0.41)**
*Per recommendation met*:	**-0.59 (-0.63, -0.55)**	**-0.15 (-0.18, -0.11)**	**-0.13 (-0.18, -0.08)**	**-0.17 (-0.22, -0.12)**
*Lifestyle based on overall adherence*	***Anxiety symptoms***			
Unfavourable	**-1.24 (-1.46, -1.01)**	-0.13 (-0.31, 0.05)	0.03 (-0.23, 0.28)	**-0.29 (-0.54, -0.03)**
Intermediate	**-2.24 (-2.48, -2.00)**	**-0.40 (-0.60, -0.20)**	-0.17 (-0.45, 0.11)	**-0.65 (-0.93, -0.38)**
Favourable	**-2.90 (-3.72, -2.08)**	-0.40 (-1.06, 0.25)	-0.54 (-1.54, 0.46)	-0.33 (-1.17, 0.51)
*Per recommendation met*:	**-0.44 (-0.48, -0.40)**	**-0.10 (-0.14, -0.07)**	**-0.07 (-0.12, -0.03)**	**-0.13 (-0.18, -0.09)**

β: unstandardized regression coefficient; 95% CI: 95% confidence interval; SSB: sugar-sweetened beverages; MVPA: moderate-to-vigorous physical activity

*Multivariable linear mixed-effects models were adjusted for age, ethnicity, weight status, weight perception, weight loss attempts, school-area median household income, school area urban class, and mental health at baseline (CESD-R-10 and GAD-7 scores, as appropriate). Meeting 3 or less recommendations was the reference category in analyses where lifestyle based on overall adherence was the independent variable. CIs that do not include the null value are bolded

**Those meeting 3 or less recommendations were classified as having very unfavourable, 4–6 – unfavourable, 7–9 – intermediate, and 10–12 – favourable lifestyles

## Discussion

In this study, we found adherence to 12 lifestyle behaviours in middle adolescence to be very low, particularly for vegetables and fruit, grains, and screen time with less than 5% of participants adhering to each of these recommendations. Participants reported adhering, on average, to 5.6 lifestyle recommendations and almost none adhering to all 12 recommendations. Adherence to recommendations for lifestyle behaviours, particularly for meat and alternatives, SSB, screen time, sleep, and no cannabis use, was prospectively associated with less severe depressive and anxiety symptoms in middle adolescents. When considered in combination, adolescents meeting 10–12 and 7–9 recommendations had lower CESD-R-10 scores and those meeting 7–9 recommendations had lower GAD-7 scores at follow-up, compared to those with the very unfavourable lifestyle (i.e., meeting ≤ 3 recommendations). Moreover, adherence to each additional recommendation was associated with lower CESD-R-10 and GAD-7 scores at follow-up.

As mentioned before, there is only one prospective study that considered individual and combined effects of adherence to a range of lifestyle behaviours in relation to mental health outcomes, but it is limited to early adolescence. This study by Loewen et al. linked population-based survey data from 3,436 early adolescents (10–11 year old) to administrative records up to the age of 15. Early adolescents meeting 4–6 and 7–9 lifestyle recommendations had, respectively, 39% and 56% fewer healthcare visits for mental health problems in the following four years compared to those meeting 1–3 lifestyle recommendations. [16] Available studies on the combined effect (i.e., the impact of overall adherence to recommendations on mental health) in *older* adolescents are cross-sectional in design. For example, a study of 10,183 adolescents in grades 7–12 showed that meeting recommendations for sleep, screen time, and physical activity was linked to lower odds of suicidal ideation (odds ratio [OR] = 0.24, 95% CI 0.09, 0.69) and suicide attempts (OR = 0.08, 95% CI 0.02, 0.41) in male students. [46] Another cross-sectional study of 244,250 Norwegian adolescents aged 13–19 showed that those with higher overall adherence to recommendations for physical activity, screen time, tobacco smoking, and alcohol consumption had up to 60% lower odds of depressive symptoms. [47] Although results reported in these studies are encouraging, prospective effect estimates are more reliable since they consider baseline levels of mental health.

Prospective estimates reported in the current study should be considered through a population health lens. As Geoffrey Rose noted, [48] ‘a large number of people at a small risk may give rise to more cases of disease than the small number who are at a high risk’. Although evidence shows that targeted prevention of mental disorders appear more effective and cost-effective than population-level prevention, [49] the latter covers not only those at increased risk of mental disorders, but serves as a protective shield for all children and adolescents. [50] Moreover, population-level prevention strategies for physical health conditions mostly target the same lifestyle behaviours (e.g., diet, physical activity, sedentary behaviour, screen time, substance use) as those aiming to prevent mental disorders, and therefore these strategies can be united under the shared framework for prevention of common mental and noncommunicable diseases. [51] If implemented throughout childhood and early adolescence, these interventions may be effective at curbing the increasing burden of both physical and mental health problems in the longer term.

The finding that females adhering to the physical activity recommendation tended to have higher depressive and anxiety symptoms is surprising. The same finding, albeit only in relation to anxiety symptoms at 1-year follow-up, was previously reported in another COMPASS study. [52] One may speculate the influence of social desirability bias: female adolescents, aware of existing recommendations to engage in physical activity, might over-report their MVPA, particularly those who are overweight. [53] If the severity of mental health symptoms is also over-reported by female adolescents, it could contribute to this spurious association. Nonetheless, it remains unclear what could explain this counterintuitive relationship, given that physical activity is one of the most effective lifestyle interventions to promote mental health in adolescents. [54] Still, it emphasizes the importance of conducting sex-based subgroup analyses to understand sex differences better.

### Limitations

Several limitations are worth considering when interpreting study findings. First, data come from a convenience sample predominantly from schools located in large population centres and areas with higher school-area median income. Thus, the study findings may not be representative of adolescents in Canada. Second, all measures were self-reported, yet social desirability and recall bias might have been at least partially negated by the anonymous nature of the COMPASS questionnaire. Third, although many measures are based on previously validated national surveillance tools and guidelines, measurement error is possible, leading to misclassification. [21] Considering the trajectories of adherence to recommendations in relation to mental health outcomes could partly overcome the measurement error associated with the self-reported adherence to lifestyle recommendations, but given this study’s short timeframe (i.e., one year), we assumed that trajectories of most lifestyle behaviours stayed fairly stable. Finally, analyses could be overpowered given the large sample size, and hence we focus on effect estimates rather than p-values in our interpretation of the results, as per current recommendations. [55, 56]

## Conclusion

In prospective analyses, we found that overall adherence to recommendations for a comprehensive set of lifestyle behaviours common in middle adolescents was associated with lower severity of depressive symptoms in females and males and anxiety symptoms in males one year later. These results support the public health message that making even modest positive changes to lifestyle behaviours that increase overall adherence to lifestyle recommendations can improve mental health in adolescents. Given the multifactorial etiology of mental health problems, population-based approaches promoting healthy lifestyle behaviours, particularly those with the lowest prevalence (e.g., healthy eating, limited screen time), may yield the biggest improvement in mental health in adolescents, along with many other health, social, and developmental outcomes.

## Electronic supplementary material

Below is the link to the electronic supplementary material.

Supplementary Material 1 Table S1. Associations of individual adherence to 12 lifestyle recommendations with the CESD-R-10 and GAD-7 scores at baseline

Supplementary Material 2 Table S2. Associations of overall adherence to 12 lifestyle recommendations with CESD-R-10 and GAD-7 scores at baseline

Supplementary Material 3 Recruitment, enrollment, retention over the follow-up period, and missing data

Supplementary Material 4 STROBE Checklist

## Data Availability

Data are available on reasonable request. COMPASS data are available for researchers upon successful completion and approval of the COMPASS data usage application (https://uwaterloo.ca/compass-system/information-researchers).

## List of abbreviations

ANOVA: analysis of variance
CESD-R-10: Center for Epidemiologic Studies Depression Scale Revised-10
COVID-19: Coronavirus disease 2019
COMPASS: Cannabis, Obesity, Mental health, Physical activity, Alcohol, Smoking, and Sedentary behaviour
GAD-7: General Anxiety Disorder-7
LMM: linear mixed-effects model
MVPA: moderate-to-vigorous physical activity
OR: odds ratio
SD: standard deviation
SSB: sugar-sweetened beverages
