# Supplementary material for: The impact of 12 modifiable lifestyle behaviours on depressive and anxiety symptoms in middle adolescence: prospective analyses of the Canadian longitudinal COMPASS study
Source: Int J Behav Nutr Phys Act. 2023 Apr 17;20:45. doi: 10.1186/s12966-023-01436-y (PMC10107579; doi:10.1186/s12966-023-01436-y)
Supplement: Supplementary file 1 — Supplementary Material 1 Table S1. Associations of individual adherence to 12 lifestyle recommendations with the CESD-R-10 and GAD-7 scores at baseline [file 12966_2023_1436_MOESM1_ESM.docx]

Table S1. Associations of individual adherence to 12 lifestyle recommendations with the CESD-R-10 and GAD-7 scores at baseline

|  | **Univariate** | **Multivariable*** | | | |
| --- | --- | --- | --- | --- | --- |
|  |  | **Model 1** | **Model 2** | | |
|  | **Total** | **Total** | **Total** | **Females** | **Males** |
|  | **β (95% CI)** | **β (95% CI)** | **β (95% CI)** | **β (95% CI)** | **β (95% CI)** |
| *Adherence to individual recommendations for:* | ***Depressive symptoms*** | | | | |
| Vegetables and fruit | 0.02 (-0.35, 0.39) | -0.07 (-0.42, 0.28) | 0.27 (-0.07, 0.62) | 0.20 (-0.27, 0.67) | 0.28 (-0.24, 0.80) |
| Grain products | -0.09 (-0.44, 0.25) | 0.24 (-0.09, 0.57) | 0.50 (0.17, 0.82) | 0.38 (-0.13, 0.90) | **0.54 (0.14, 0.94)** |
| Milk and alternatives | **-0.88 (-1.03, -0.73)** | **-0.25 (-0.40, -0.10)** | -0.09 (-0.23, 0.05) | -0.07 (-0.29, 0.15) | -0.12 (-0.30, 0.06) |
| Meat and alternatives | **-0.40 (-0.54, -0.25)** | **-0.85 (-0.99, -0.71)** | **-0.73 (-0.87, -0.59)** | **-1.11 (-1.31, -0.90)** | **-0.24 (-0.42, -0.06)** |
| SSB | **-0.90 (-1.06, -0.74)** | **-0.93 (-1.08, -0.77)** | **-0.51 (-0.66, -0.36)** | **-0.61 (-0.83, -0.40)** | **-0.39 (-0.59. -0.19)** |
| MVPA | **-0.49 (-0.64, -0.34)** | -0.02 (-0.16, 0.13) | -0.02 (-0.16, 0.12) | **0.21 (0.00, 0.43)** | **-0.25 (-0.43, -0.07)** |
| Screen time | **-1.83 (-2.16, -1.49)** | **-1.73 (-2.04, -1.41)** | **-1.19 (-1.50, -0.88)** | **-1.26 (-1.68, -0.84)** | **-0.86 (-1.32, -0.41)** |
| Sleep | **-2.35 (-2.49, -2.20)** | **-1.91 (-2.05, -1.76)** | **-1.70 (-1.84, -1.56)** | **-1.85 (-2.05, -1.64)** | **-1.54 (-1.72, -1.36)** |
| No tobacco smoking | **-3.06 (-3.34, -2.77)** | **-2.94 (-3.22, -2.67)** | **-1.47 (-1.78, -1.16)** | **-1.97 (-2.45, -1.49)** | **-1.18 (01.56, -0.79)** |
| No vaping | **-1.69 (-1.88, -1.49)** | **-1.76 (-1.95, -1.58)** | **-0.48 (-0.70, -0.27)** | **-0.83 (-1.15, -0.50)** | -0.27 (-0.54, 0.01) |
| No cannabis use | **-2.65 (-2.86, -2.44)** | **-2.46 (-2.67, -2.26)** | **-1.24 (-1.48, -1.00)** | **-1.56 (-1.92, -1.20)** | **-0.85 (-1.16, -0.53)** |
| No binge drinking | **-1.79 (-1.96, -1.62)** | **-1.56 (-1.73, -1.39)** | **-0.43 (-0.62, -0.24)** | **-0.57 (-0.85, -0.29)** | -0.08 (-0.33, 0.16) |
| *Adherence to individual recommendations for:* | ***Anxiety symptoms*** | | | | |
| Vegetables and fruit | **0.43 (0.09, 0.77)** | 0.25 (-0.07, 0.58) | **0.45 (0.13, 0.77)** | **0.47 (0.04, 0.90)** | 0.29 (-0.20, 0.78) |
| Grain products | 0.09 (-0.23, 0.41) | **0.44 (0.14, 0.74)** | **0.56 (0.25, 0.86)** | 0.44 (-0.04, 0.91) | **0.62 (0.25, 1.00)** |
| Milk and alternatives | **-0.77 (-0.91, -0.63)** | -0.11 (-0.24, 0.02) | -0.03 (-0.16, 0.11) | 0.06 (-0.14, 0.27) | -0.11 (-0.28, 0.06) |
| Meat and alternatives | -0.03 (-0.16, 0.11) | **-0.58 (-0.71, -0.45)** | **-0.53 (-0.66, -0.40)** | **-0.82 (-1.09, -0.63)** | -0.12 (-0.29, 0.05) |
| SSB | **-0.79 (-0.94, -0.65)** | **-0.85 (-0.99, -0.71)** | **-0.55 (-0.69, -0.42)** | **-0.62 (-0.82, -0.42)** | **-0.46 (-0.65, -0.28)** |
| MVPA | **-0.42 (-0.56, -0.28)** | 0.09 (-0.04, 0.23) | 0.07 (-0.06, 0.20) | **0.28 (0.09, 0.48)** | -0.15 (-0.32. 0.02) |
| Screen time | **-1.06 (-1.37, -0.76)** | **-1.08 (-1.37, -0.79)** | **-0.66 (-0.94, -0.37)** | **-0.66 (-1.05, -0.28)** | **-0.52 (-0.95, -0.09)** |
| Sleep | **-1.76 (-1.89, -1.62)** | **-1.36 (-1.49, -1.23)** | **-1.20 (-1.33, -1.08)** | **-1.40 (-1.59, -1.22)** | **-0.97 (-1.14, -0.80)** |
| No tobacco smoking | **-2.24 (-2.50, -1.97)** | **-2.18 (-2.43, -1.93)** | **-1.06 (-1.34, -0.77)** | **-1.46 (-1.90, -1.02)** | **-0.80 (-1.16, -0.44)** |
| No vaping | **-1.23 (-1.41, -1.05)** | **-1.33 (-1.50, -1.16)** | **-0.34 (-0.54, -0.14)** | **-0.65 (-0.95, -0.35)** | -0.14 (-0.40, 0.11) |
| No cannabis use | **-2.00 (-2.20, -1.81)** | **-1.81 (-2.00, -1.63)** | **-0.86 (-1.09, -0.64)** | **-1.01 (-1.34, -0.68)** | **-0.69 (-0.99, -0.40)** |
| No binge drinking | **-1.50 (-1.65, -1.34)** | **-1.24 (-1.39, -1.09)** | **-0.41 (-0.58, -0.23)** | **-0.48 (-0.74, -0.22)** | -0.19 (-0.42, 0.04) |

β: unstandardized regression coefficients; 95% CI: 95% confidence interval; SSB: sugar-sweetened beverages; MVPA: moderate-to-vigorous physical activity.

*In linear mixed-effect models, Model 1 was adjusted for age, ethnicity, weight status, weight perception, weight loss attempts, school- area median household income, and school area urban class. Model 2 was mutually adjusted for other lifestyle behaviours. Not meeting recommendations was the reference category in all analyses presented in Table S1. CIs that do not include the null value are bolded.
