# Supplementary material for: The impact of 12 modifiable lifestyle behaviours on depressive and anxiety symptoms in middle adolescence: prospective analyses of the Canadian longitudinal COMPASS study
Source: Int J Behav Nutr Phys Act. 2023 Apr 17;20:45. doi: 10.1186/s12966-023-01436-y (PMC10107579; doi:10.1186/s12966-023-01436-y)
Supplement: Supplementary file 2 — Supplementary Material 2 Table S2. Associations of overall adherence to 12 lifestyle recommendations with CESD-R-10 and GAD-7 scores at baseline [file 12966_2023_1436_MOESM2_ESM.docx]

Table S2. Associations of overall adherence to 12 lifestyle recommendations with CESD-R-10 and GAD-7 scores at baseline

|  | **Univariate** | | **Multivariable*** | | |
| --- | --- | --- | --- | --- | --- |
|  | **Total** | **Total** | | **Females** | **Males** |
|  | **β (95% CI)** | **β (95% CI)** | | **β (95% CI)** | **β (95% CI)** |
| *Lifestyle based on overall adherence*** | ***Depressive symptoms*** | | | | |
| Unfavourable | **-2.32 (-2.55, -2.09)** | **-2.11 (-2.33, -1.88)** | | **-2.69 (-3.02, -2.37)** | **-1.37 (-1.66, -1.08)** |
| Intermediate | **-4.03 (-4.27, -3.78)** | **-3.56 (-3.80, -3.32)** | | **-4.44 (-4.79, -4.08)** | **-2.49 (-2.81, -2.17)** |
| Favourable | **-4.95 (-5.79, -4.11)** | **-3.99 (-4.81, -3.19)** | | **-5.31 (-6.62, -4.01)** | **-2.76 (-3.73, -1.79)** |
| *Per recommendation met:* | -0.76 (-0.80, 0.72) | **-0.67 (-0.71, -0.63)** | | **-0.85 (-0.91, -0.79)** | **-0.47 (-0.52, -0.42)** |
| *Lifestyle based on overall adherence* | ***Anxiety symptoms*** | | | | |
| Unfavourable | **-1.87 (-2.09, -1.66)** | **-1.64 (-1.84, -1.43)** | | **-2.04 (-2.34, -1.75)** | **-1.12 (-1.39, -0.85)** |
| Intermediate | **-3.09 (-3.32, -2.86)** | **-2.66 (-2.88, -2.43)** | | **-3.26 (-3.59, -2.94)** | **-1.89 (-2.19, -1.60)** |
| Favourable | **-3.86 (-4.64, -3.08)** | **-2.92 (-3.67, -2.18)** | | **-4.10 (-5.28, -2.91)** | **-1.87 (-2.77, -0.97)** |
| *Per recommendation met:* | **-0.56 (-0.60, -0.53)** | **-0.48 (-0.52, -0.44)** | | **-0.60 (-0.66, -0.55)** | **-0.33 (-0.38, -0.29)** |

β: unstandardized regression coefficients; 95% CI: 95% confidence interval; SSB: sugar-sweetened beverages; MVPA: moderate-to-vigorous physical activity.

*Multivariable linear mixed-effects models were adjusted for age, ethnicity, weight status, weight perception, weight loss attempts, school- area median household income, and school area urban class. Meeting 3 or less recommendations was the reference category in analyses where lifestyle based on overall adherence was the independent variable. CIs that do not include the null value are bolded.

**Those meeting 3 or less recommendations were classified as having very unfavourable, 4-6 – unfavourable, 7-9 – intermediate, and 10-12 – favourable lifestyles.
