# Supplementary material for: The impact of 12 modifiable lifestyle behaviours on depressive and anxiety symptoms in middle adolescence: prospective analyses of the Canadian longitudinal COMPASS study
Source: Int J Behav Nutr Phys Act. 2023 Apr 17;20:45. doi: 10.1186/s12966-023-01436-y (PMC10107579; doi:10.1186/s12966-023-01436-y)
Supplement: Supplementary file 3 — Supplementary Material 3 Recruitment, enrollment, retention over the follow-up period, and missing data [file 12966_2023_1436_MOESM3_ESM.docx]

Supplementary Material 3

**Recruitment and enrollment**

The Cannabis, Obesity, Mental health, Physical activity, Alcohol, Smoking, and Sedentary behaviour (COMPASS) is a large longitudinal study that annually collects survey data on an extensive range of lifestyle behaviours from more than 65,000 grade 9-12 students (age 13-18 years). Participants are recruited through a convenience sample of 122 secondary schools in Alberta, British Columbia, Ontario, and Québec, Canada. As mentioned in the limitations section, most of these schools are located in large population centres and areas with higher school-area median income, thus results of this study might not be representative of adolescents in Canada. Using an active-information passive-consent parental permission protocol, students complete an anonymous COMPASS questionnaire at school during class time.

**Retention over the follow-up period**

In COMPASS study, response rates in 2017/18 and 2018/19 were 81.8% and 84.2%, respectively, with non-response being mainly due to absenteeism or scheduled spare time during data collection.

**Eligibility criteria**

For this study, we linked participants' responses from 2017/18 (average age of participants 14.8 [SD=1.2] years old) to the same participants' responses from 2018/19 (average age 15.8 [SD=1.2] years old). Records of grade 12 participants and those who changed schools between 2017/18 and 2018/19 were not linked: out of 66,434 participants from 122 schools in 2017/18, the linked sample comprised records of 29,022 participants from 116 schools. Analyses were based on a subsample of 24,274 participants, with data available for all variables listed below.

**Missing data**

Missing baseline values for sex, ethnicity, and age were imputed based on the available information (e.g., age reported in 2018/19 minus one, and ethnicity and sex same as reported in 2018/19). Additionally, values were mean imputed for 5168 participants who were missing up to two responses on CESD-R-10 and/or GAD-7 scales. Other than that, no imputation for other variables was done. Analyses were based on a subsample of 24,274 participants, with data available for all variables considered in analyses.
